# Genetic Factors Linking Nucleolar Stress with *R2* Retrotransposon Expression in *Drosophila melanogaster*

**DOI:** 10.3390/ijms26125480

**Published:** 2025-06-07

**Authors:** Shova Pandey, An Tri Nguyen, Audrey K. Maricle, Patrick J. DiMario

**Affiliations:** Department of Biological Sciences, Louisiana State University, Baton Rouge, LA 70803, USA; shova.pandey@lsu.edu (S.P.); angu283@lsu.edu (A.T.N.); amaric2@lsu.edu (A.K.M.)

**Keywords:** *R2* retrotransposon, *Drosophila*, Nopp140, nucleolar stress, rDNA, nucleolus

## Abstract

*R2* retrotransposons reside exclusively within the *28S* regions of 10–20% of all rDNA genes comprising the nucleolar organizer loci on the X and Y chromosomes of *Drosophila melanogaster*. These *R2*-inserted genes are normally silent and heterochromatic. When expressed, however, the *R2* transcript is co-transcribed with the *28S* rRNA. Self-cleavage releases a 3.6 kb mature *R2* transcript that encodes a single protein with endonuclease and reverse transcriptase activities that facilitate *R2* element transposition by target-primed reverse transcription. While we know the molecular details of *R2* transposition, we know little about the genetic mechanisms that initiate *R2* transcription. Here, we examine *R2* expression in wild type versus mutant backgrounds. *R2* expression in stage 1–4 wild type egg chambers was variable depending on the stock. *R2* expression was silent in wild type stages 5–10 but was consistently active during nurse cell nuclear breakdown in stages 12–13 regardless of the genetic background. Massive *R2* expression occurred in stages 5–10 upon loss of Udd, an RNA Pol I transcription factor. Similarly, loss of Nopp140, an early ribosome assembly factor, induced *R2* expression more so in somatic tissues. Interestingly, over-expression of the Nopp140-RGG isoform but not the Nopp140-True isoform induced *R2* expression in larval somatic tissues, suggesting Nopp140-RGG could potentially affect rDNA chromatin structure. Conversely, *Minute* mutations in genes encoding ribosomal proteins had minor positive effects on *R2* expression. We conclude that *R2* expression is largely controlled by factors regulating RNA Pol I transcription and early ribosome assembly.

## 1. Introduction

*R2* elements are non-LTR retrotransposons found in arthropods, cnidarians (corals and jellyfish), tunicates (ascidians such as sea squirts), platyhelminthes (flatworms), and vertebrates except for mammals. They have been well studied in insects for over 40 years [1,2,3,4] *R2* elements and another non-LTR retrotransposon, *R1*, preferentially insert into the *28S* regions of rDNA genes [5]. In *Drosophila melanogaster*, an *R2* element inserts at 2651 bps downstream of the start of the *28S* region, while *R1* inserts 74 bps further downstream. Approximately 44% of the *Drosophila* rDNA units contain only *R1* elements, another 11% contain only *R2* elements, and about 5% contain both *R1* and *R2* elements, although these percentages can vary between different fly stocks. *R2* and/or *R1*-inserted rDNA genes are generally silent, while uninserted rDNA genes transcribe most of the rRNA needed for ribosome production.

Fefelova et al. [6] showed that *R2*-inserted rDNA genes enrich with Heterochromatin Protein 1a (HP1a) and the H3K9me3 mark, establishing the normally repressed state for *R2*-inserted rDNA genes. When expressed, however, the *R2* element is co-transcribed with the *28S* rRNA by RNA polymerase I (Pol I), after which the 5′ end of the *R2* transcript self-cleaves to liberate itself from the *28S* rRNA [7]. Processing at the 3′ end to generate the functional *R2* transcript of 3.6 kb has not been clarified. The *R2* transcript encodes a single protein with reverse transcriptase and DNA endonuclease activities, both of which are necessary for target-primed reverse transcription and genome integration [2].

Fefelova et al. [6] also showed a massive induction of *R2* transcription in *Drosophila* egg chamber nurse cells upon loss of Under-developed (Udd), an RNA Polymerase I (Pol I) transcription factor unique to Diptera [6,8]. Udd was first characterized by Zhang et al. [8]; wild type Udd is a small protein of 18 kDa that interacts with TAF1B, TAF1C-like, Tif-1A, and Rpl135, which is one of the two largest RNA Pol I subunits. These proteins establish a transcription pre-initiation complex resembling human Selectivity factor 1 (SL1) that recruits RNA Pol I to rDNA core promoters [9]. How *R2* is co-transcribed with the *28S* rRNA in large amounts by RNA Pol I upon Udd loss remains a perplexing question. Further complicating the question, Zhang et al. [8] showed that cells homozygous for the *udd^Null^* mutant displayed reduced rRNA synthesis.

Our lab showed earlier that deleting the *Nopp140* gene in *Drosophila* induced *R2* expression [10]. The *Nopp140* gene in *Drosophila melanogaster* produces two protein isoforms that are identical up to amino acid residue 584, after which they differ in their carboxyl tails due to alternative splicing [11,12]. We refer to the isoforms as Nopp140-True, a close (true) orthologue to vertebrate Nopp140 [13], and Nopp140-RGG with a carboxyl tail closely resembling the carboxyl tail of Nucleolin, an abundant ribosome assembly factor in vertebrates [14]. Similar RGG/RG domains constitute an intrinsically disordered RNA and/or protein binding domain in many other RNA-associated proteins [15,16]. Interestingly, *Drosophila* lacks a close orthologue to vertebrate Nucleolin, while vertebrates lack a Nopp140-RGG isoform. The single vertebrate isoform of Nopp140 is believed to participate in both rRNA Pol I transcription and pre-rRNA processing [17].

Nucleolar stress results from failures to provide enough ribosomes, while ribosomal stress occurs when the ribosomes that are produced contain a mutated ribosomal protein [18,19]. We proposed that the loss of Nopp140 and thus ribosome production induces nucleolar stress in *Drosophila* [10]. We predicted that a feedback mechanism would respond by activating the normally silent *R2*-inserted rDNA genes to compensate for the loss of ribosome production. However, we did not observe *R1* expression upon loss of Nopp140, suggesting that the expression of *R2* versus *R1* elements is differentially regulated. We later showed that *R1* could be induced by heat shock while *R2* remained silent [20].

The loss of nucleolar proteins known or predicted to regulate RNA Pol I transcription (Udd and Nopp140) should reduce ribosome production (cause nucleolar stress), while mutations of individual ribosomal proteins should reduce ribosome function, thus creating *Minute* phenotypes (e.g., cause ribosomal stress). Here, we tested several genetic backgrounds associated with either nucleolar stress or ribosomal stress for the induction of *R2* expression. In general, we saw induced *R2* expression with the loss of the Pol I regulators, Udd and Nopp140, while the loss of ribosomal proteins had reduced effects on *R2* expression. These observations support the hypothesis that *R2* expression is controlled by factors that regulate Pol I transcription. Interestingly, while over-expression of both Nopp140 isoforms impaired rRNA transcription, we saw robust *R2* expression when we over-expressed the Nopp140-RGG isoform but not the Nopp140-True isoform. The observation suggests a putative disruption in nucleolar chromatin mediated by excessive Nopp140-RGG.

## 2. Results

### 2.1. R2 Expression in Wild Type Ovaries—A Complex Negative Control

We used fluorescence in situ hybridization (FISH) to detect *R2* transcripts in adult female egg chambers as an indicator for nucleolar stress. Wild type nurse cells produce on the order of 1 × 10^10^ ribosomes [21], which are eventually donated to the developing egg through inter-connecting ring canals. Because of this high ribosome production rate and based on earlier work [6,10], we reasoned that any perturbation in ribosome production would induce *R2* expression in these egg chambers.

**Figure 1:**
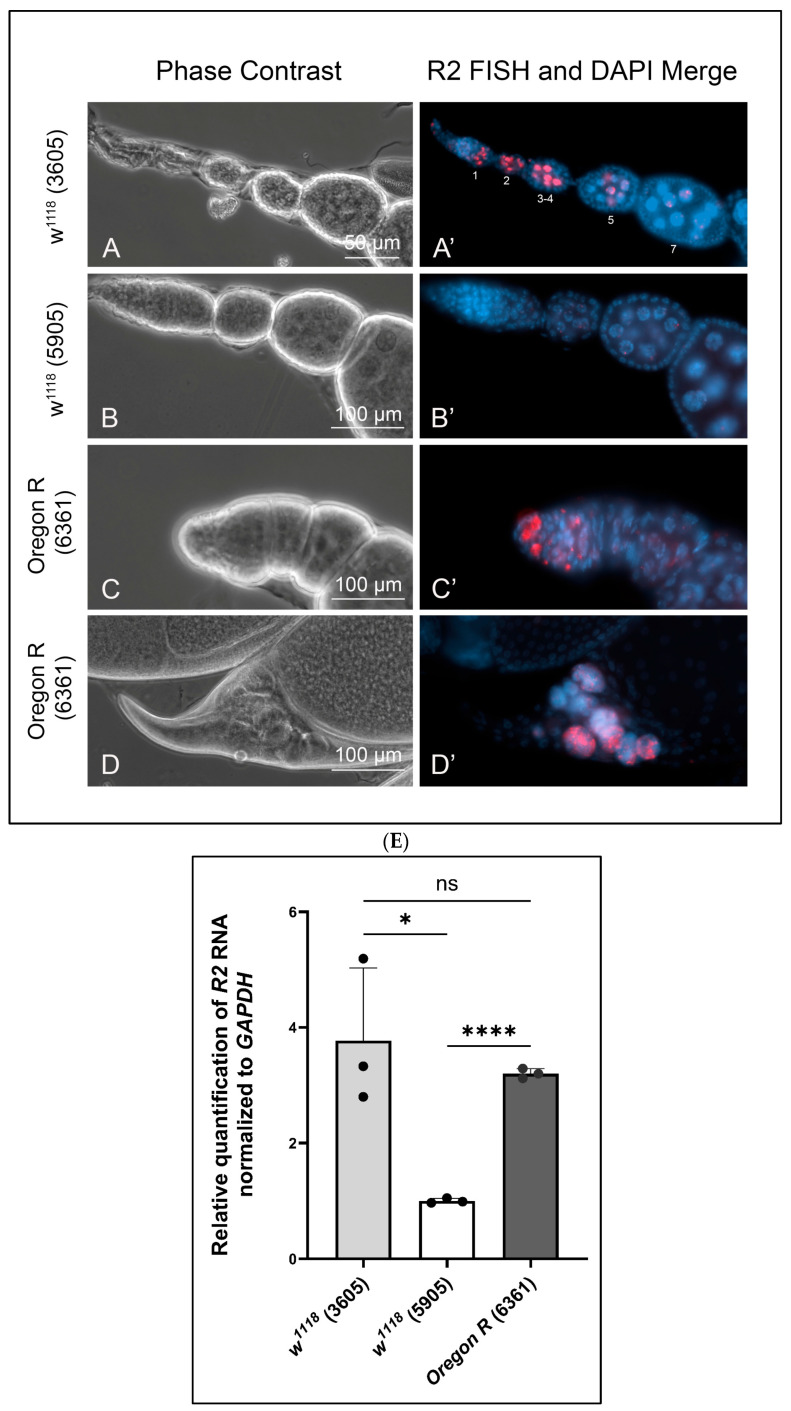
Fluorescence in situ hybridizations showing variable *R2* expression in wild type egg chambers. (**A**,**A′**): *R2* was detected in egg chamber stages 1 through 4 (staging by [21]) from *w^1118^* (stock 3605). *R2* was nearly depleted by stages 5 and 7. (**B**,**B′**): *R2* expression was barely detectable in *w^1118^*, stock 5905. (**C**,**C′**): *R2* was detected in 2–3 large cells in the most anterior regions of the germarium in Oregon R (stock 6361) ovaries. (**D**,**D′**): *R2* expression was consistently detected in stage 12 and 13 remnant nurse cell nuclei as nurse cells degraded by apoptosis. (**E**): RT-qPCR measured the expression levels of *R2* in whole ovary normalized to *GAPDH*. Bloomington stock numbers are in parentheses. Error bars indicate mean ± standard deviation of three replicates for each fly line, and statistical significance was calculated by an unpaired Student’s *t*-test using GraphPad Prism 10.2.0 with *p*-values < 0.1 as (*), *p*-values < 0.0001 as (****), and ns refers to not significant.

To establish a baseline for *R2* expression in wild type egg chambers, we tested three lines: *w^1118^* (stock 3605), *w^1118^* (stock 5905 with isogenized chromosomes 1, 2, and 3), and Oregon R (stock 6361). Initially, we thought *R2* expression would be negligible, as the germline is known to suppress transposable element expression and mobilization due to the expression of Piwi-interacting RNAs (piRNAs) [22,23]. However, we were surprised to detect reproducible *R2* expression in the *w^1118^* stock 3605 (Figure 1A,A′) and in the Oregon R stock, 6361 (Figure 1C,C′). Expression in *w^1118^* stock 3605 was in region 3 of the germarium (i.e., a stage 1 egg chamber) but not in germarium regions 1 and 2 (Figure 1A,A′). *R2* expression was maintained in nurse cell nuclei in stages 2–4 but then declined in stage 5 egg chambers. Interestingly, this pattern of *R2* expression coincides with the polytenic stages of nurse cell chromosomes as they undergo initial rounds of endoreplication. In stage 5, the chromosomes continue to endoreplicate, but they do not condense back into blob-like polytene structures as seen in the early stages. Instead, the chromosomes disperse giving rise to the multi-lobed nucleolar structures necessary for rapid ribosome production [21,24,25].

On the other hand, the isogenic *w^1118^* stock 5905 showed negligible levels of *R2* expression in the germarium and young egg chambers (Figure 1B,B′). *R2* expression in the Oregon R stock 6361 was confined to 2–3 large cells in region 1 of the germarium (Figure 1C,C′). Presumably, these are germline stem cells or cystoblasts. *R2* expression was absent in Oregon R germarium regions 2 and 3 and in egg chambers through stage 10. Taken together, these observations indicate variable *R2* expression levels in wild type germaria and young egg chambers in a stock-dependent manner. We do not know what genetic factors contribute to this variability.

While *R2* expression was silent through stages 5–10 for all three wild type stocks, its expression re-occurred reproducibly in stages 12 and 13 in all three stocks (Figure 1D,D′). Nurse cells normally dump their cytoplasmic contents into the oocyte through inter-connecting ring canals beginning in stage 11. Nurse cells then begin to degrade in stage 12 via apoptosis, autophagy, and phagocytosis [26]. Nuclear breakdown within these nurse cells occurs by apoptosis beginning in stage 12 and continues into stage 13. *R2* expression was evident in the residual nurse cell nuclear material that normally remains outside the oocyte in stages 12 and 13 (Figure 1D,D′).

While FISH provides a cell-by-cell comparison of R2 expressions, we also used RT-qPCR to compare *R2* expression levels in whole ovaries from the three wild stocks (Figure 1E). Values for *R2* RNA levels were normalized to values for transcripts encoding GAPDH. This allowed us to compare these wild type values to those from various mutant ovaries. Stock 5905 (*w^1118^*) showed the least *R2* expression compared to stock 3605 (*w^1118^*) and stock 6361 (Oregon R), but *R2* expression levels ranged from 1- to 4-fold in ovaries from the three stocks. These values were negligible compared to *R2* expression levels found in ovaries from various mutant backgrounds described below.

Since *R2* elements map to the nucleolar organizer regions on the X and Y chromosomes, we kept track of the various X chromosome pairs in females used for all subsequent RT-qPCR assays presented below (see Table 1). We found no correlation between various X chromosome pairs and the extent of *R2* expression. In other words, *R2* expression is better correlated with the mutation disrupting ribosome biogenesis rather than to variabilities between different X chromosomes pairs.

### 2.2. Udd Mutations Affecting R2 Expression—A Clear Positive Control

**Figure 2:**
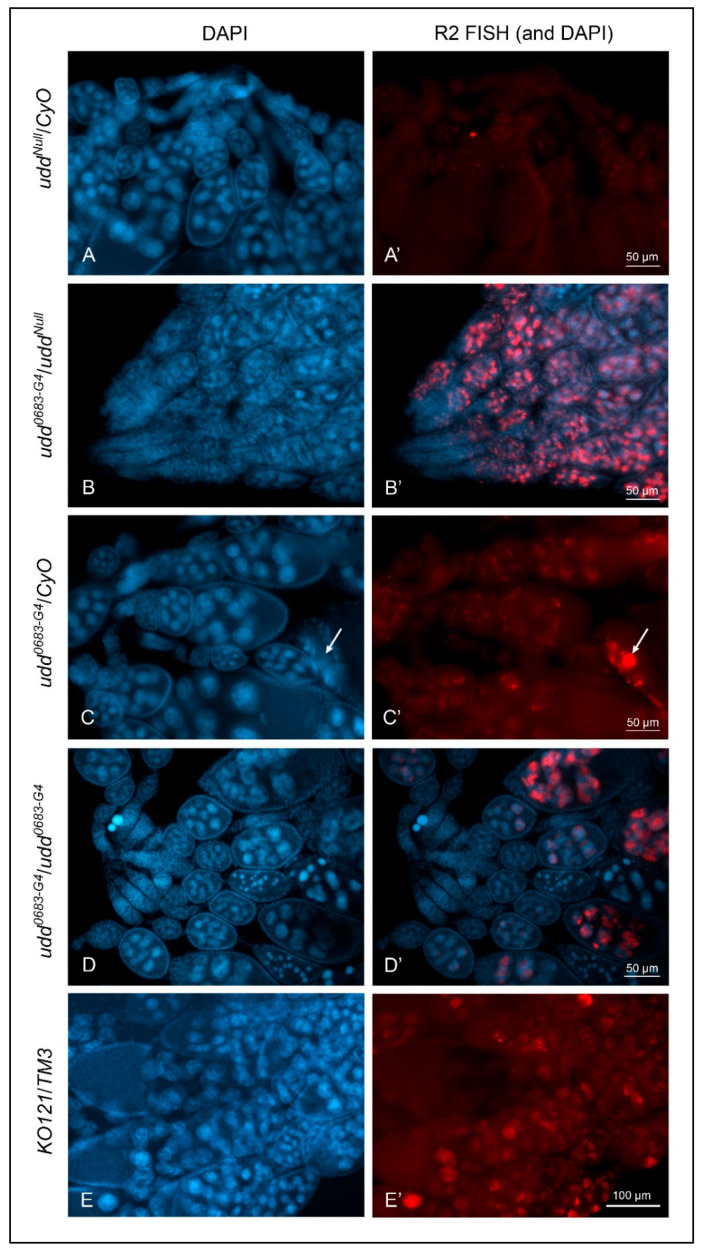

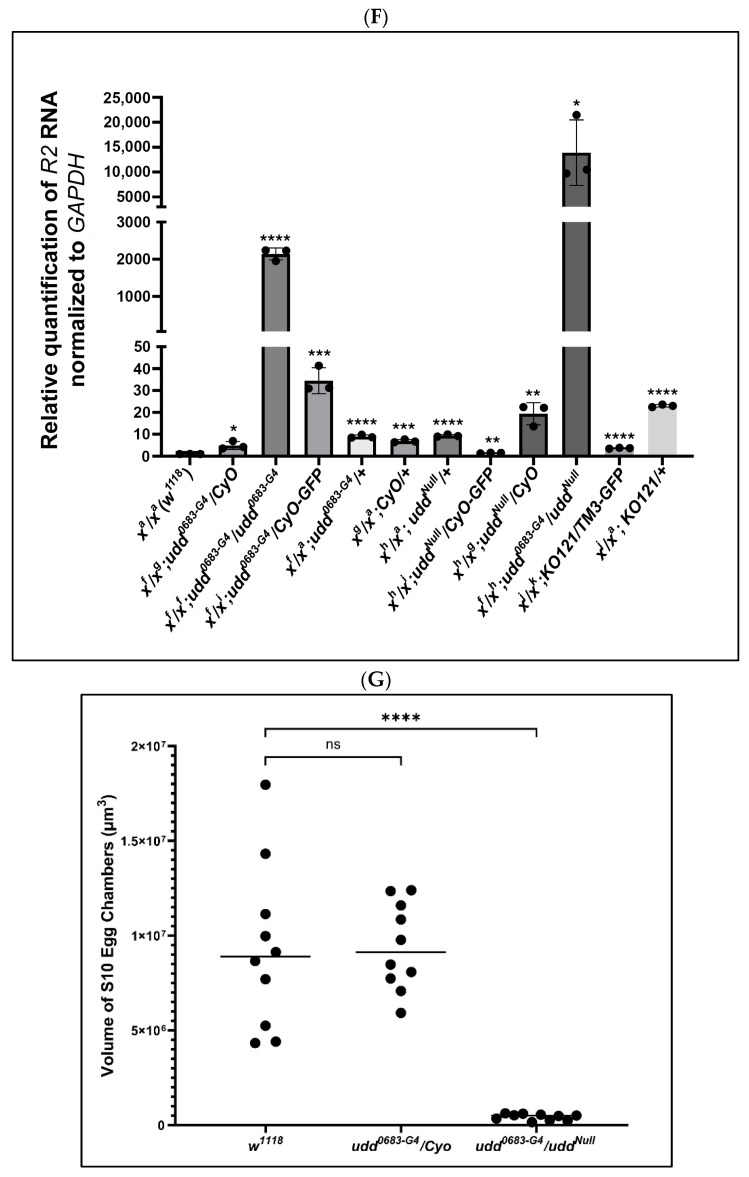
Loss of Udd was a positive control for *R2* expression as detected by FISH. (**A**,**A′**): Ovaries from *udd^Null^/CyO* females showed no appreciable *R2* expression. (**B**,**B′**): Ovaries from *udd^0683-G4^/udd^Null^* females showed abundant *R2* expression but in a reciprocal manner from than that shown for ovaries from *w*^1118^ females (Figure 1A′) with no expression in early egg chambers, copious amounts in intermediate chambers, and diminished expression in the older egg chambers. (**C**,**C′**): Ovaries from *udd^0683-G4^/CyO* females were generally devoid of *R2* expression except for stage 12–13 residual nurse cell chromatin (white arrow) as seen in *w^1118^* ovaries. (**D**,**D′**): Ovaries from *udd^0683-G4^/udd^0683-G4^* females showed *R2* expression in older stage 10 egg chambers. (**E**,**E′**): *R2* expression in *Nop140* gene knockout *KO121* balanced over *TM3*. *R2* was detected sporadically in nurse cell nuclei. (**F**): RT-qPCR measurements for *R2* transcripts in ovaries isolated from various *udd* and *Nopp140* mutations. X chromosome pairs are designated for the females. Error bars show mean ± standard deviation of three replicates for each fly line. Statistical significance was determined by an unpaired Student’s *t*-test using GraphPad Prism 10.2.0 with *p*-values < 0.1 indicated as (*), *p*-values < 0.01 as (**), *p*-values < 0.001 as (***), and *p*-values < 0.0001 as (****). (**G**): The volume of stage 10 egg chambers from *w^1118^*, *udd^0683-G4^/CyO,* and *udd^0683-G4^/udd^Null^* females were determined using a formula for an ellipsoid. Stage 10 egg chambers from *udd^0683-G4^/udd^Null^* females were approximately 20-fold smaller in volume than the volume of stage 10 chambers from their sibling *udd^0683-G4^/CyO* sisters. Statistical analysis was done by an unpaired Student’s *t*-test using GraphPad Prism 10.2.0 with *p*-value < 0.0001 indicated as (****). No statistical difference: ns.

Fefelova et al. [6] showed substantial *R2* expression in egg chambers from *udd^0683-G4^/udd^Null^* females. Udd is one of several proteins constituting the Selectivity factor 1 (SL1)-like complex in *Drosophila*. In mammals, SL1 recruits RNA Polymerase I to core rDNA promoters. While *udd^Null^/CyO* egg chambers showed little if any *R2* expression (Figure 2A,A′), *udd^0683-G4^/udd^Null^* egg chambers showed extremely high levels of *R2* expression in nurse cell nuclei of stage 4/5–10 egg chambers (Figure 2B,B′). This is contrary to what we saw in all three wild type egg chambers (Figure 1). Nurse cells from *udd^0683-G4^/CyO* sibling females showed very low *R2* expression levels (Figure 2C,C′); the one bright spot in Figure 2C′ (arrow) represents the apoptotic nurse cell nuclear material in a stage 12–13 egg chamber as described for similarly staged wild type egg chambers in Figure 1D. Egg chambers from homozygous *udd^0683-G4^* females also showed substantial *R2* expression (Figure 2D,D′) but not nearly to the extent as found in *udd^0683-G4^/udd^Null^* egg chambers.

Figure 2F provides RT-qPCR values for *R2* expression in adult ovaries from the various *udd* and *Nopp140* mutant genotypes. Values for *R2* RNA levels were again normalized to values for *GAPDH* transcripts. For each genotype, we kept track of the two X chromosomes in the respective females, as well as the balancer chromosomes that could potentially affect *R2* expression. The RT-qPCR results correlated well with the FISH observations of Figure 2A–D: *R2* expression was significantly higher in ovaries from *udd^0683-G4^/udd^0683-G4^* and *udd^0683-G4^/udd^Null^* females but relatively low in the intermediate stocks used throughout the course in this work. (*R2* expression in egg chambers and whole ovaries from females heterozygous for the *Nopp140* gene deletion (*KO121/TM3*; Figure 2E,E′,F) is described below).

The *udd^0683-G4^/udd^Null^* egg chambers (Figure 2B,B′) appeared reduced in size by light microscopy. Assuming stage 10 egg chambers are ellipsoids, their volumes can be approximated using the equation V = 4/3·(π)·*a·b·c*, where *a* is the radius of the egg chamber’s length, and values *b* and *c* are the radii at right angles to each other at the widest points of the egg chamber. We assumed the two width radii are equal. Using these assumptions, the volume of stage 10 egg chambers from *udd^0683-G4^/CyO* measured ~9 × 10^6^ µm^3^ on average and appeared normal in size as compared to wild type egg chambers. On the other hand, the volume of *udd^0683-G4^/udd^Null^* stage 10 egg chambers measured ~4 × 10^5^ µm^3^ (Figure 2G). Thus, the *udd^0683-G4^/CyO* stage 10 egg chambers were ~23 times greater in volume than the *udd^0683-G4^/udd^Null^* stage 10 egg chambers.

**Figure 3:**
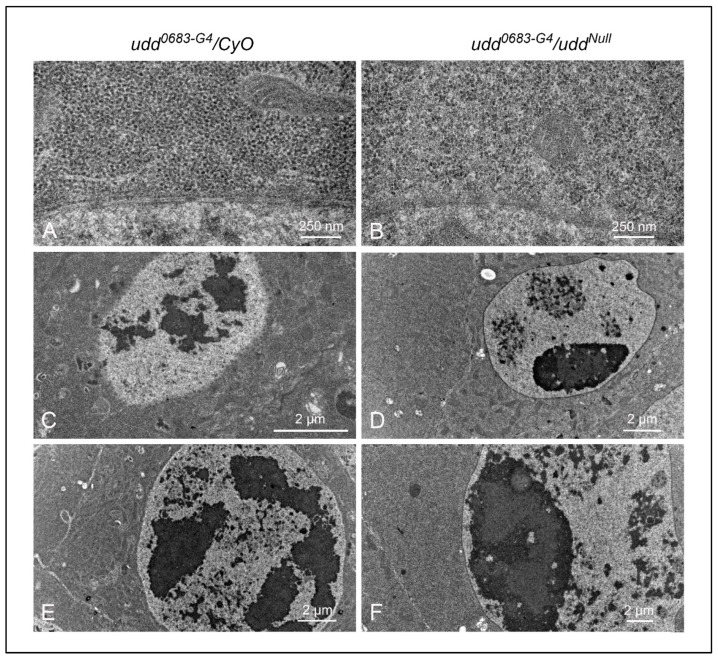

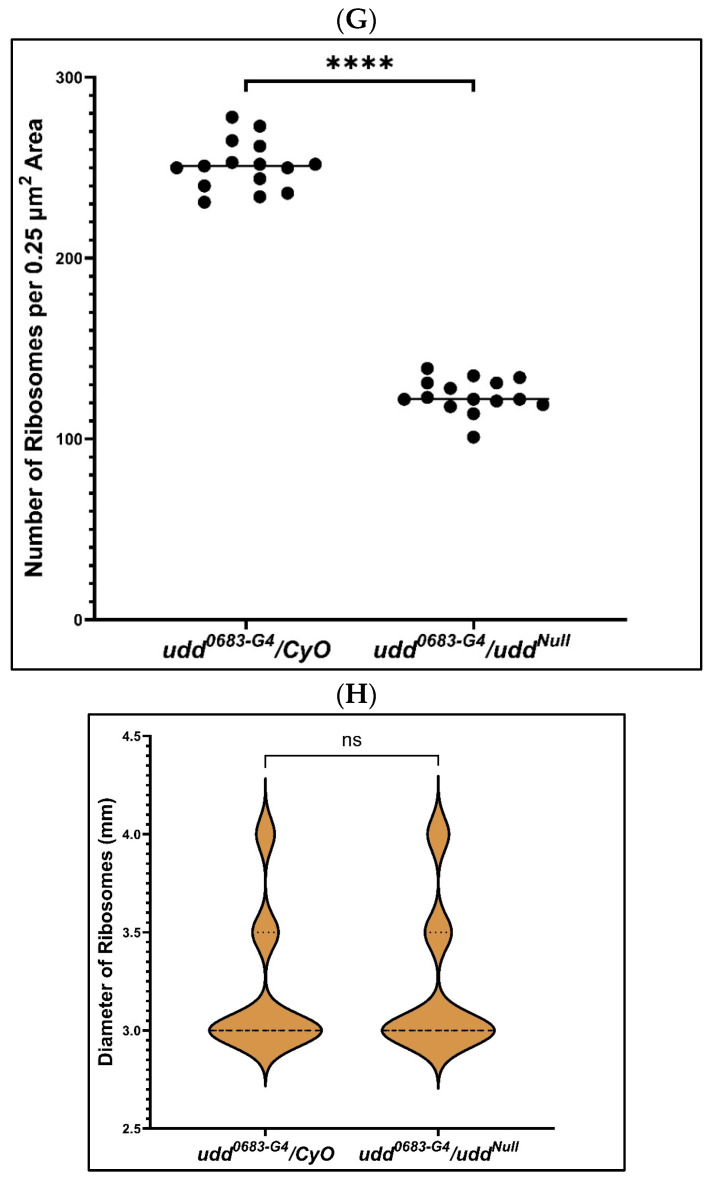
Ultrastructural examination of egg chambers from *udd^0683-G4^/CyO* females (**A**,**C**,**E**) and *udd^0683-G4^/udd^Null^* females (**B**,**D**,**F**). (**A**,**B**): a visual comparison of cytoplasmic ribosome abundance and distribution between *udd^0683-G4^/CyO* and *udd^0683-G4^/udd^Null^* nurse cells (also see panel (**G**)). (**C**,**D**): Nucleolar structure appears normal in younger *udd^0683-G4^/CyO* egg chambers, while *udd^0683-G4^/udd^Null^* nurse cells generally contain one prominent nucleolus with smaller nucleolar-like patches with what appeared to be partial activity. The one large nucleolus should be producing copious amounts of *R2*. (**E**,**F**): An older *udd^0683-G4^/CyO* egg chamber shows a normal distribution of multiple nucleolar lobes while the *udd^0683-G4^/udd^Null^* nurse cell showed one large nucleolus with ultrastructural sub-regions. (**G**): Ribosome counts per unit area (25 µm^2^) were taken from images like those in panels (**A**,**B**). *udd^0683-G4^/udd^Null^* nurse cells contained half the number of ribosomes per unit area compared to *udd^0683-G4^/CyO* nurse cells. n = 15, where n is the total number of unit areas selected for each genotypic line. An unpaired Student’s *t*-test using GraphPad Prism 10.2.0 with *p*-value < 0.0001 indicated as (****) was implied to calculate the statistical significance. (**H**) There was no difference observed in the diameters of ribosomes in *udd^0683-G4^/CyO* versus *udd^0683-G4^/udd^Null^* nurse cells (number of ribosomes measured for each = 60).

We next crossed *udd^Null^/CyO* females to homozygous *udd^0683-G4^* males and prepared ovaries from sibling *udd^0683-G4^/CyO* and *udd^0683-G4^/udd^Null^* females to assess ribosome density and ultra-structure of their nurse cells and nucleoli (Figure 3). Ribosome densities in the nurse cells of vitellogenic egg chambers from *udd^0683-G4^/CyO* females (Figure 3A) appeared normal [21,27]. We counted about 250 ribosomes per 0.25 µm^2^ (Figure 3A,G), while ribosome densities in nurse cells of vitellogenic chambers from *udd^0683-G4^/udd^Null^* females were reduced (Figure 3B,G) to about 125 ribosomes per 0.25 µm^2^. Factoring in the reduced volume of the *udd^0683-G4^/udd^Null^* egg chambers further indicates a substantial reduction in total ribosome production in the *udd^0683-G4^/udd^Null^* nurse cells as compared to *udd^0683-G4^/CyO* nurse cells. Finally, we measured ribosome size in both *udd^0683-G4^/CyO* and *udd^0683-G4^/udd^Null^* egg chambers but saw no difference (Figure 3H) in their size suggesting the ribosomes that are present in *udd^0683-G4^/udd^Null^* egg chambers are fully assembled.

In terms of nucleolar ultra-structure, the *udd^0683-G4^/CyO* nurse cells from pre-vitellogenic egg chambers (Figure 3C) and vitellogenic egg chambers (Figure 3E) displayed normal nucleolar lobes typical of nucleoli in wild type nurse cells [28]. These lobes each contained a central region surrounded by darker, more peripheral regions that we interpret to be dense fibrillar components (DFCs) and granular regions (GRs), respectively. Conversely, *udd^0683-G4^/udd^Null^* nurse cells in pre-vitellogenic and vitellogenic egg chambers (Figure 3D,F, respectively) contained one large nucleolus with discernible DFCs but with more extensive GRs. Besides the one prominent nucleolus in the *udd^0683-G4^/udd^Null^* nucleus in Figure 3D, there were 2–3 other nuclear regions that likely represented nucleolar lobes with partial rDNA synthetic activity as denoted by vastly reduced RNP deposits.

To summarize, egg chambers from *udd^0683-G4^/udd^Null^* females expressed copious amounts of *R2* RNA (Figure 2D,F), were substantially in reduced size (Figure 2G), and contained half the number of ribosomes per unit area when compared to egg chambers from their sibling *udd^0683-G4^/CyO* sisters (Figure 3G). These egg chambers failed to establish multiple, productive nucleolar lobes, consistent with failures in ribosome production.

### 2.3. R2 Expression in Tissues Depleted for Nopp140

Nopp140 is considered an early ribosome assembly factor that likely interacts with RNA Pol I [29,30,31]. As with the loss of Udd, loss of Nopp140 likely disrupts early ribosome biogenesis at the level of Pol I transcription (chromatin). Earlier, we showed abundant *R2* expression levels in larvae homozygous for a *Nopp140* gene deletion (*KO121)* [10]. Specifically, Northern blot analysis of total larval RNA showed abundant accumulations of the self-cleaved 3.6 kb *R2* transcript in homozygous *KO121* larvae as compared to control larvae. We speculated at the time [10] that loss of Nopp140 and thus ribosome production might induce a compensatory mechanism within cells to activate normally silent rDNA genes, many of which contain *R2* elements.

When homozygous, the *KO121* deletion is lethal in the second larval instar stage. We maintain the deletion using the *TM3* balancer chromosome. While heterozygous adults are viable and fertile, we have noted a low rate of embryonic lethality in the heterozygotes (unpublished) suggesting these individuals experience some degree of nucleolar stress (a haplo-insufficiency). We therefore used FISH to test if *R2* elements were expressed in *KO121/TM3* egg chambers due to partial nucleolar stress. We found *R2* expressed in the larger, more posterior nurse cells, but the fluorescence intensities overall were lower and more variable than those found in *udd^0683-G4^/udd^Null^* egg chambers (compare Figure 2B,B′ with Figure 2E,E′). RT-qPCRs likewise showed a rather modest 10-fold elevation in *R2* levels in ovaries from heterozygous *KO121/TM3* females compared to *w^1118^* ovaries (Figure 2F).

**Figure 4:**
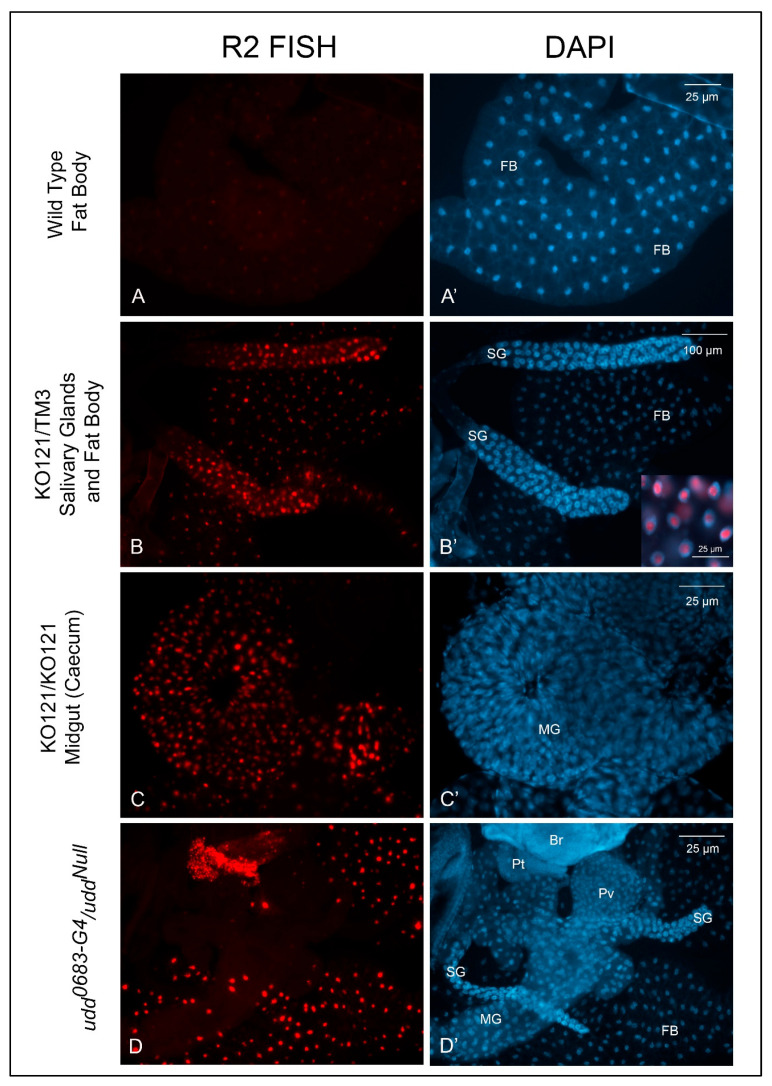
*R2* expression in somatic tissues from different genotypes. (**A**,**A′**): *R2* expression was not evident in *w^1118^* larval or adult somatic tissues. (**B**,**B′**): *KO121/TM3* larval tissues showed detectable *R2* expression with heavier expression in the larval salivary glands. The inset in panel (**B′**) shows the overlay of FISH and DAPI labeling. *R2* transcripts enriched within the nucleoli. (**C**,**C′**): Heavy *R2* expression was apparent in most larval somatic tissues from homozygous *KO121* larvae. (**D**,**D′**): Somatic tissues from *udd^0683-G4^/udd^Null^* larvae also expressed heavy amounts of *R2*. Br, brain; FB, fat body; MG, midgut; Pt, prothoracic gland; Pv, proventriculus; SG, salivary gland.

We also used somatic tissues to explore *R2* expression levels by FISH. While Figure 4A,A′ shows only fat body, *R2* expression was typically absent in wild type larval tissues. *R2* expression, however, was prominent in several tissues from *KO121/TM3* larvae as (Figure 4B,B′). Midgut tissues from still-living *KO121*/*KO121* second instar larvae (Figure 4C,C′) and several different tissues from *udd^0683-G4^/udd^Null^* larvae also showed substantial *R2* expression levels as expected. While we have not quantified these *R2* expression levels in somatic tissues by RT-qPCR, the FISH results verify *R2* expression occurred in various tissues, and this helps establish a haplo-insufficiency of the *Nopp140* gene in *KO121/TM3* larvae.

### 2.4. Over-Expression of Nopp140-RGG but Not Nopp140-True Induces R2 Expression

**Figure 5:**
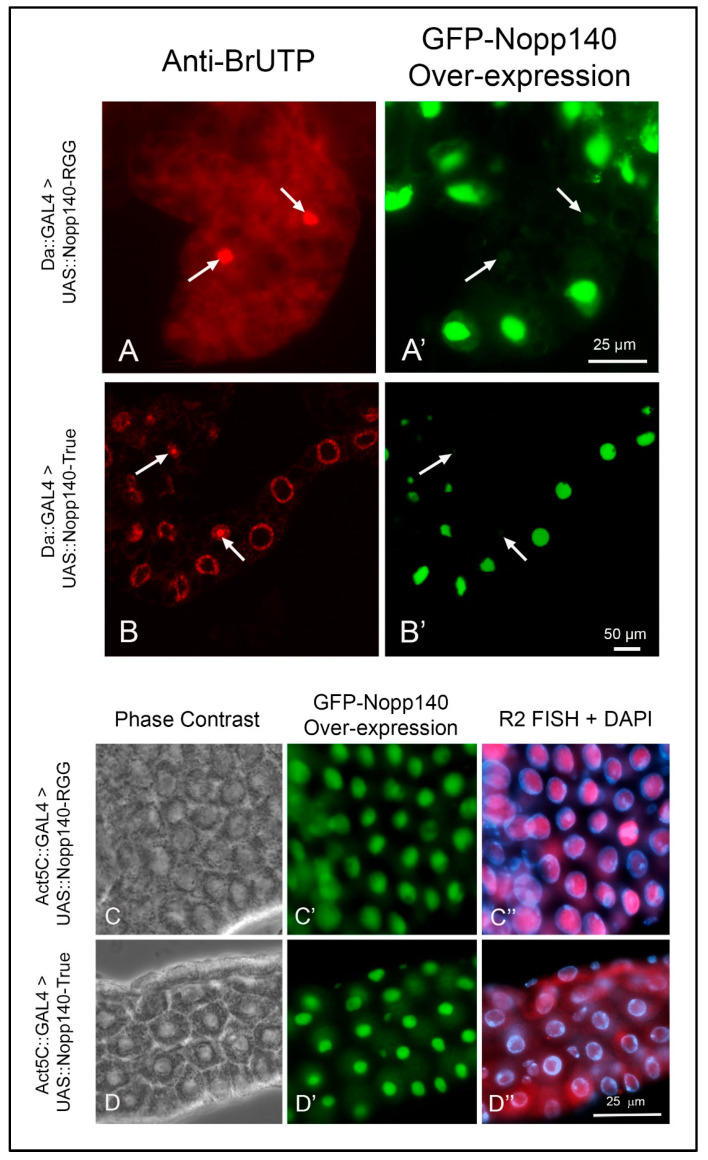

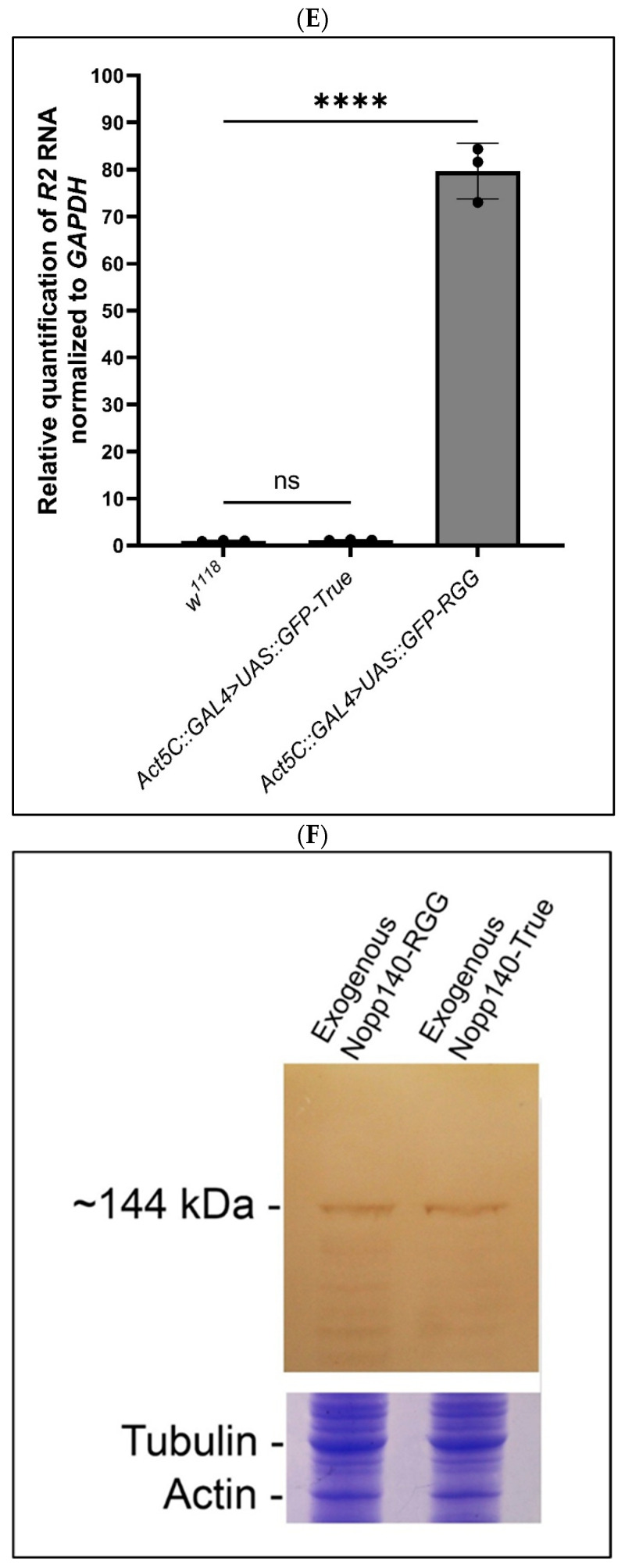
Over-expression of Nopp140-RGG but not Nopp140-True induces *R2* expression in tissues pulse-labeled with BrUTP. (**A**,**A′**): *UAS::GFP::Nopp140-RGG* (fly stock G3) was induced using the *Da::GAL4* driver, which typically fails to express in all cells (arrows). Conventional fluorescence microscopy showed fat body cells that failed to over-express Nopp140-RGG incorporated BrUTP normally into their nucleoli, while enlarged nucleoli in neighboring cells that over-expressed Nopp140-RGG failed to label with BrUTP. (**B**,**B′**): *Da-GAL4>UAS::GFP::Nopp140-True* (fly stock A12) showed similarly enlarged nucleoli by confocal microscopy of Malpighian tubules. Again, no BrUTP labeling was detected in the swollen nucleoli but rather in chromosomal regions flanking the nucleoli. (**C**–**C″**): *UAS::GFP::Nopp140-RGG* (fly stock H1) was induced using the *Act5C::GAL4* driver. Midgut tissue expressed *R2* transcripts that enriched with the swollen nucleoli. (**D**–**D″**): *UAS::GFP::Nopp140-True* (fly stock A9) was induced using the *Act5C::GAL4* driver. Nucleoli were again swollen but very little if any *R2* transcripts accumulated in these nucleoli. (**E**): RT-qPCRs validated the *R2* expression levels in larvae for nucleolar stress induced due to over-expression of Nopp140-RGG. Error bars depict mean ± standard deviation of three replicates for each fly line and statistical significance was calculated by an unpaired Student’s *t*-test using GraphPad Prism 10.2.0 with *p*-values < 0.0001 indicated as (****). No statistical difference: ns. (**F**): An immunoblot probed with anti-GFP showing similar expression levels for GFP-Nopp140-RGG and GFP-Nopp140-True from transgenic *UAS* lines H1 and A9, respectively. The Nopp140 isoforms normally migrate at ~120 ka, but with the GFP tag, they were at ~144 kDa. A companion SDS gel was stained with Coomassie Blue to show similar loads for the two samples.

We over-expressed the two Nopp140 isoforms, Nopp140-RGG and Nopp140-True, as GFP-tagged fusions from *UAS* transgenes using either the *Da::GAL4* or *ACT5C::GAL4* drivers. For unknown reasons, the *Da::GAL4* transgene typically does not express in all cells, and we took advantage of this as these few cells failed to over-express Nopp140. These non-expressing cells served as internal controls. Dissected tissues were pulse-labeled with BrUTP for 30 min, washed (chased) for 10 min, and then fixed and probed with anti-BrUTP. Cells that failed to over-express the isoforms maintained normal nucleolar morphology, and they incorporated BrUTP normally into their nucleoli (arrows in Figure 5A,B). Conversely, cells that over-expressed Nopp140-RGG (Figure 5A,A′) or Nopp140-True (Figure 5B,B′) showed enlarged nucleoli and failed to incorporate BrUTP into these nucleoli. We conclude that the separate over-expression of both Nopp140 isoforms disrupted nucleolar morphology and blocked the apparent synthesis of rRNA in the swollen nucleoli.

We next used FISH and RT-qPCR to determine if *R2* was expressed due to apparent nucleolar stress caused by over-expressing the two Nopp140 isoforms. FISH showed *R2* transcripts enriched within the swollen nucleoli of midgut cells from *Act5C::GAL4>UAS::GFP-Nopp140-RGG* larvae (Figure 5C–C″). Conversely, midgut cells from *Act5C::GAL4>UAS::GFP-Nopp140-True* larvae showed very little if any *R2* transcripts in the swollen nucleoli (Figure 5D–D″).

RT-qPCR supported the FISH results; there was an 80-fold accumulation of *R2* in the *Act5C::GAL4>UAS::GFP-Nopp140-RGG* larvae compared to the *Act5C::GAL4>UAS::GFP-Nopp140-True* larvae (Figure 5E). Both genotypes produced comparable amounts of UAS-induced GFP-fusion protein (Figure 5F). We conclude that the RGG carboxyl tail is likely responsible for the observed *R2* induction (see the Discussion for a possible explanation). How *R2* is expressed with an overall loss of nucleolar transcription is the same perplexing question encountered with *R2* expression upon loss of Udd.

### 2.5. Testing R2 Expression in Minute Genetic Backgrounds

**Figure 6:**
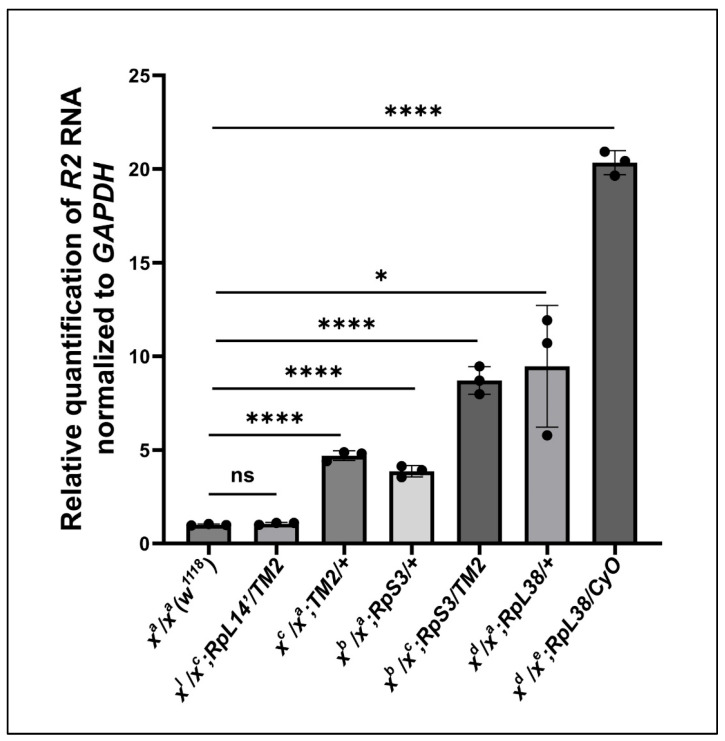
RT-qPCRs measured *R2* abundance in ovaries from females heterozygous for *Minute* mutations. Error bars represent the mean ± standard deviation of three replicates for each fly line. Statistical analysis was determined by an unpaired Student’s *t*-test using GraphPad Prism 10.2.0 with *p*-values < 0.1 indicated as (*) and *p*-values < 0.0001 as (****). No statistical difference: ns.

Up to this point, we have attributed the expression of *R2* to the loss of Udd and both Nopp140 isoforms (gene deletion) or to the over-expression of only Nopp140-RGG. Both Udd and Nopp140 function in very early ribosome biogenesis (Pol I transcription). If a compensatory mechanism exists to activate normally silent rDNA genes upon loss of ribosome production, this mechanism may also link the loss of ribosome function with *R2* induction. To begin testing this, we used *Minute* mutations that in *Drosophila* reside in genes encoding individual ribosomal proteins [32]. We examined *R2* expression levels in *larvae* heterozygous for the *Minute* mutations, RpS3^2^, *RpL14^1^, and RpL38^45-72^.* All three *Minute* mutations are dominant, they display adverse *Minute* phenotypes in adults (FlyBase), and they are embryonic lethal when homozygous. As such, stocks are maintained using balancer chromosomes. *R2* expression in adult ovaries from these three heterozygous mutations is relatively low as measured by RT-qPCRs (Figure 6), reaching a maximum fold induction of 20 over wild type levels. Thus, while abundant *R2* expression depends on failures in early ribosome biogenesis at the level of Pol I transcription (chromatin), individual *Minute* mutations as heterozygotes have only minor positive effects on *R2* induction.

## 3. Discussion

### 3.1. An Overview

We previously showed substantial *R2* expression upon deletion of the *Nopp140* gene in *Drosophila* [10]. Here, we compared *R2* expression in egg chambers upon loss of Udd or Nopp140 (Figure 2) with some initial analyses showing subtle variations in *R2* expression in egg chambers of different wild type stocks (Figure 1). Figure 3 carefully shows the loss of ribosome production in egg chambers lacking Udd. The nucleolar ultrastructure in Figure 3 helps establish the Udd null phenotype. Figure 4 showed *R2* expression in somatic tissues lacking Udd or Nopp140. This is critical to understand *R2* expression upon Nopp140 loss, as the *Nopp140* gene deletion is larval lethal when homozygous, and it helps establish *Nopp140* as haplo-insufficient. Figure 5 presented a novel finding showing that *R2* is induced upon over-expression of the Nopp140-RGG isoform but not the Nopp140-True isoform. As explained below, the combined observations of Figure 1A and Figure 5 provide a testable hypothesis for *R2* regulation based on RGG/RG-containing proteins regulating the formation/maintenance of nucleolar heterochromatin. Our initial goal in this study was to test if *R2* is induced upon nucleolar or ribosomal stress. Clearly, *R2* was induced when early ribosome biogenesis factors (Udd and Nopp140) were mutated/deleted. However, unlike the loss of Udd or Nopp140, we saw minimal effects by the *Minute* mutations tested here (Figure 6), suggesting that ribosomal stress may not be a primary inducer for *R2* expression.

### 3.2. R2 Expression in Wild Type Ovaries

During oogenesis, *Drosophila* nurse cells produce vast amounts of maternal ribosomes that eventually pass into the rapidly expanding egg. Thus, we chose to assess *R2* expression initially in egg chambers upon nucleolar stress. We assumed that wild type egg chambers would serve as a negative control for *R2* expression, as Piwi is known to suppress expression of transposable elements (i.e., *R2*) in the female germ line. We were surprised, therefore, to see *R2* expression in stage 1–4 egg chambers from homozygous *w^1118^* females (Bloomington stock 3605, Figure 1A,A′) and in region 1 of germaria in ovaries from the Oregon R females (stock 6361, Figure 1C,C′). We did not see *R2* expression in early egg chambers from the isogenic *w^1118^* females (stock 5905, Figure 1B,B′). We note that *R2* expression in these wild type stocks was negligible by RT-qPCR when compared to experimental stocks lacking Udd or Nopp140; the abscissa ranging from 0–4 in Figure 1E is directly comparable to the abscissa in Figure 2F.

*R2* expression was suppressed in egg chamber stages 5–10 for all three wild type stocks, as expected. Yet, its expression typically appeared in residual nurse cell nuclei that remained outside the oocyte in stages 12 and 13 (Figure 1D,D′). Nurse cell chromatin degrades normally by apoptosis in stages 12 and 13, as the nurse cell cytoplasm dumps into the oocyte through ring canals. We speculate that *R2* elements are briefly activated upon loss of restrictive heterochromatin marks or the loss of general heterochromatin structure, for example, as HP1a degrades by apoptosis.

### 3.3. Does a Compensation Mechanism Exist to Activate R2-Inserted rDNA Genes?

Nelson et al. [33] showed that *R2* activation is critical for the restoration of rDNA copy numbers in the *Drosophila* male germline (referred to as rDNA magnification). These authors proposed that the *R2* endonuclease is essential for rDNA expansion, as it creates double-stand breaks at the rDNA genes, resulting in rDNA recombination and rDNA copy number expansion. Indeed, *R2* expression appears higher in cells when rDNA copy numbers are reduced [33], suggesting a compensation mechanism that induces normally silent rDNA genes with inserted *R2* elements.

Besides a loss of rDNA units, other mutations known to induce *R2* expression include the loss of Udd, TAF1B, TAF1C, and Heterochromatin Protein 1a (HP1a) [6]. In *Drosophila*, Udd interacts with TAF1B and TAF1C to form a Selectivity Factor 1-like complex [8], which is necessary for recruiting RNA polymerase I (Pol I) to initiate pre-rRNA transcription. Zhang et al. [8] reported that loss of Udd reduced Pol I transcription (reduced BrUTP incorporation) as well as the reduced steady state amounts of pre-rRNA and pre-rRNA processing intermediates. They also reported an accumulation of RpS2-GFP within nucleoli upon loss of Udd. Thus, all indications suggest that *R2* up-regulation occurs at the level of rDNA and RNA Pol I transcription [2].

We examined *udd^0683-G4^/udd^Null^* nurse cells by TEM and observed a substantial reduction (by about 50%) in ribosome content per unit area (Figure 3B,G). These *udd^0683-G4^/udd^Null^* nurse cells also maintained just one large functional nucleolus compared to the multi-lobed nucleolar structures normally present in wild type nurse cells (Figure 3D,F). These single nucleoli contained expanded GRs, which could explain the reported accumulation of RpS2-GFP [8]. The *udd^0683-G4^/udd^Null^* females survived to adulthood, indicating their ribosome production must be adequate for somatic tissues throughout development. However, the single large nucleolus in *udd^0683-G4^/udd^Null^* nurse cells is likely inadequate for producing the huge stockpile of maternal ribosomes needed for oogenesis and embryogenesis. These females fail to lay eggs.

Nopp140 is considered an early ribosome assembly factor, but immuno-precipitation assays by others indicated that mammalian Nopp140 also associates with RNA Pol I [29,30,31]. Like the loss of Udd, the deletion of *Nopp140* causes a substantial loss of ribosome production and an induction of *R2* expression [10]. As shown by FISH in this study, loss of Nopp140 in homozygous *KO121* somatic larval tissues resulted in substantial *R2* expression (Figure 4C,C′), comparable to the FISH signals in somatic tissues from *udd^0683-G4^/udd^Null^* larvae (Figure 4D,D′). Perhaps the large induction of normally silent *R2*-inserted rDNA genes in *udd^0683-G4^/udd^Null^* and *KO121/KO121* tissues is an attempt by the cell to compensate for the overall loss in pre-rRNA production. If such a compensatory mechanism exists, it likely occurs at the chromatin level, but it remains unknown.

### 3.4. Developing a Hypothesis for R2 Induction

Interestingly, *R2* expression in the early egg chambers of *w^1118^* stock 3605 appeared to correlate with the normal maturation in nurse cell chromatin [18,24,25,34]. For instance, *R2* expression in stages 1–4 egg (Figure 1A′) tracks closely with the polytenic state of the nurse cell chromosomes. We know that nucleolar expansion (increased ribosome production) occurs in these nurse cells after DNA endoreplication cycle 5 (stages 4–5), just when we saw *R2* expression decline (Figure 1A′). In this chromosome expansion process, the one nucleolus commonly found in nurse cells of stage 1–4 egg chambers disperses, producing the expanded nucleolar lobes that we typically see in nurse cells of later-stage egg chambers. Khipple and King [34] noted that nucleolar expansion fails when chromosome dispersion is blocked after endocycle 5 in ovaries homozygous for the recessive *fs(1)1304* mutation, now referred to as *small ovary* (*sov*). Nucleolar expansion appeared to likewise fail in the small egg chambers from *udd^0683-G4^/udd^Null^* females (Figure 3D,F).

More recently, Jankovics et al. [35] proposed that the wild type Sov protein maintains the germline stem cell niche by regulating heterochromatin formation and transposon silencing. Sov is a large protein of 3313 amino acids that recruits HP1a to the chromatin. Most interesting, the first half of Sov consists of RGG/RG repeats while the second half contains 21 zinc fingers and a (-PxVxL-) domain known to interact with HP1a. Jankovics et al. [35] suggested Sov interacts with both HP1a and perhaps piRNAs within chromatin to suppress transposon expression. The two Nopp140 isoforms in *Drosophila* differ only in their carboxyl ends. Perhaps by over-expressing Nopp140-RGG but not Nopp140-True (Figure 5E), we interfere with Sov’s role in maintaining heterochromatic structure ultimately resulting in *R2* induction.

Recent interest in RGG/RG domains lies in their propensity to form liquid phase condensates [15,36] not only under stress conditions [37] but now also in chromatin structure [35]. Like mammalian Nopp140, we see associations between the Pol I transcription machinery and both versions of *Drosophila* Nopp140 in ectopic nucleoli formed in vivo (Pandey and DiMario, in preparation). One possibility that we are pursuing is that both Nopp140 isoforms serve as a molecular link between RNA Pol I transcription and early pre-rRNA processing (early ribosome assembly), analogous to the long carboxyl terminal domain (CTD) of RNA Pol II and pre-mRNA. If both versions of Nopp140 associate with the Pol I complex, the question arises, does the RGG domain simply bind pre-rRNA in early ribosome assembly as we traditionally thought, or can it interact with piRNAs as suggested for the Sov protein [35] to perhaps regulate nucleolar chromatin structure and thus *R2* expression upon nucleolar stress?

## 4. Materials and Methods

### 4.1. Fly Stocks

Fly stocks were kept at 18 °C or 22–24 °C (room temperature) on standard cornmeal-molasses medium. Stock numbers are those of the Bloomington Stock Center (Indiana University, Bloomington, IN, USA). Wild type stocks included Oregon R (#6361), *w^1118^* (#3605), and *w^1118^* (#5905 with isogenized chromosomes 1, 2, and 3). *Minute* mutations included RpS3^2^/TM2 (#1696), *RpL14^1^/TM2* (#2247), and *RpL38^45-72^/CyO* (#6974). The *udd^0683-G4^* mutation was Bloomington stock #63478. The *udd^Null^*/*CyO* stock (more precisely, *Df(2R)Exele00152-d08197*) was a gift from Michael Buszczak [8]. GAL4 drivers included *Da::GAL4* (#8641) and *Act5C::GAL4* (#4414). Our over-expressing *UAS::GFP::Nopp140-RGG* cDNA fly lines were G3 and H1, while our over-expressing *UAS::GFP::Nopp140-True* cDNA fly lines were A9 and A12. These lines were described by Cui and DiMario [38]. The *Nopp140* gene deletion (*KO121/TM3*) was described by He et al. [10]. We used FlyBase (release August 20, 2024) for information on gene function and expression, cDNA clones, and fly stocks [39].

### 4.2. HCR^TM^ RNA-FISH Hybridization

HCR^TM^ RNA-FISH was performed as described [40] with some modifications. The *Drosophila R2* retrotransposon probe sets, amplifiers, hybridization buffer, probe wash buffer, and amplification buffer were purchased from Molecular Instruments (Los Angeles, CA, USA, molecularinstruments.org accessed on 5 June 2024). Details of the probe are shown in Table 2. Ovaries from adult females (*n* = 6 to 10) were dissected directly in 700 µL of PBSTT (1 × phosphate-buffered saline with 0.1% Tween-20, 0.3% Triton X-100, and 0.4% sodium azide) plus 4% paraformaldehyde (PFA) diluted from freshly prepared 10% PFA. Tissues were fixed for about 45 min at room temperature and washed four times in 5 min intervals with 700 µL of PBST (1 × PBS with 0.1% Tween-20 and 0.4% azide) by gentle shaking at room temperature. Tissues were then incubated in 700 µL of 2 µg/mL of proteinase K in PBST for 5 min at room temperature followed by three washes for 5 min each with 700 µL of PBST by gentle shaking at room temperature. Post-fixation of tissues was performed for 5 min using 700 µL of PBST containing 4% paraformaldehyde followed by six washes at 2 min intervals with 700 µL of PBST by gentle shaking at room temperature. Probe hybridization buffer and PBST were used in 1:1 ratio at room temperature to rinse the tissues. Pre-hybridization used 200 µL of hybridization buffer for 30 min at 37 °C. After pre-hybridization, 200 µL of hybridization solution containing 16 nM of the *R2Dm* probe was added and tissues were incubated overnight (>17 h) at 37 °C. The next day, tissues were washed six times with 700 µL probe wash buffer for 15 min each at 37 °C and three times with 700 µL of 5 × SSCT (5 × sodium chloride, sodium citrate, and 0.1% Tween-20) for 5 min each at room temperature. Tissues were incubated in 700 µL of amplification buffer for 10 min at room temperature. Hairpin H1 and hairpin H2 solutions, each at 6 pmol were prepared separately by incubating at 95 °C for 90 s and then allowing them to cool to room temperature in the dark for 30 min. The amplification buffer was replaced by the hairpin solution that contained the mixture of cooled H1 and H2 solutions in 100 µL of amplification buffer and incubated overnight (>17 h) by gentle shaking in the dark at room temperature. The next day, tissues were washed with 700 µL of 5 × SSCT at room temperature in the following order: 2 × 5 min, 2 × 30 min, and 1 × 5 min. Before the final wash, tissues were stained with DAPI and preserved on microscope slides with ProLong^TM^ anti-fade mounting medium (Invitrogen, Waltham, MA, USA). Samples were viewed using either an in-lab Zeiss Axioskop or a Leica SP8 confocal microscope in the Shared Instrumentation Facility (SIF) at Louisiana State University (LSU).

### 4.3. BrUTP Labeling

FuGene 6 Transfection Reagent (Roche (Basel, Switzerland), cat. no. 1815091) was used to introduce BrUTP (Sigma (St. Louis, MO, USA), cat. no. B7166) into living *Drosophila* larval tissues to label nascent RNA. Prior to dissecting the larvae, an aliquot of FuGene 6 was diluted 1/10 using dissection buffer A (15 mM HEPES, pH 6.8, 80 mM KCl, 16 mM NaCl, 5 mM MgCl_2_, and 1% Polyethylene Glycol [PEG] 6000). The dilution was left to stand at room temperature for 5 min. Lyophilized BrUTP was dissolved in MilliQ water to 100 mM. An aliquot was then added to the FuGene 6 solution such that the final BrUTP concentration was 10 mM; this solution was again left to stand at room temperature for 15 min. During this 15 min incubation, larvae that over-expressed either GFP-Nopp140-RGG (*Da-GAL4>UAS::GFP::Nopp140-RGG*, fly stock G3) or GFP-Nopp140-True (*Da-GAL4>UAS::GFP::Nopp140-True*, fly stock A12) were dissected in buffer A. Internal tissues were well exposed to the surrounding solution, which was then drawn off with a heat-pulled Pasteur pipette. The FuGene 6/BrUTP solution was then added immediately, and tissues were pulse-labeled for 30 min with gentle shaking. Tissues were then chased for 10 min with fresh buffer A lacking FuGene 6/BrUTP. The tissues were fixed with 2% paraformaldehyde in 1 × PBS, pH 7.2, and prepared for immuno-fluorescence microscopy according to de Cuevas and Spradling [41]. We used mouse anti-BrUridine (BioLegend (San Diego, CA, USA), cat. no. 317901) diluted 1/200 and Alexa Fluor 546 anti-mouse (Molecular Probes, Eugene, OR, USA) diluted 1/400 to detect the incorporated BrUridine.

### 4.4. RT-qPCR

Ten pairs of ovaries were dissected in 1 × PBST and homogenized in 1 mL TRIzol (Invitrogen). RNA isolation and precipitation were performed using Zymo-Spin II CR columns according to the manual. We used PCR to check if samples contained contaminating intronic DNA. Final RNA amounts were determined using NanoDrop 2000 (Thermo Scientific, Waltham, MA, USA). RT-qPCR was performed with a QuantStudio 6 instrument and a Luna Universal One-step RT-qPCR Kit (New England Biolabs (Ipswich, MA, USA) #E3005) with 500 ng of purified RNA template for each sample. The RT-qPCR target expression was normalized to the glycolytic enzyme, glyceraldehyde 3-phosphate dehydrogenase (GAPDH) mRNA expression, and compared with wild type (*w^1118^*) levels. Primers for the RT-qPCRs are listed in Table 2. To measure *R2* levels in Nopp140 over-expression or deletion lines, about 60 larvae 3 days after egg laying (AEL) were collected, and total RNA was isolated using TRIzol reagent and Zymo-Spin II CR columns as described.

### 4.5. Microscopy and Ribosome Counts

Most fluorescence microscopy was performed using an in-lab Zeiss Axioskop equipped with a SPOT Pursuit black and white digital camera. Images were colorized and size-calibrated using the SPOT 5.2 software. Adult ovaries were prepared for transmission electron microscopy as described [42]. We used a JEOL 1400 TEM operating at 120 KV and equipped with Gatan’s Orius digital camera in the SIF at LSU. We used Photoshop 6.1 (Adobe, San Jose, CA, USA) to prepare images for publication. To count ribosomes, we prepared 0.25 µm^2^ areas on several high-resolution TEM micrographs originally captured at 100,000× (Figure 3A,B) and simply counted recognizable ribosomes within the unit area.

### 4.6. Western Blotting

Thirty *w^1118^* larvae, 30 *Act5C::GAL4>UAS::GFP::Nopp140-RGG* (fly stock H1) larvae, or 30 *Act5C::GAL4>UAS::GFP::Nopp140-True* (fly stock A9) larvae were collected from juice plates, washed free of yeast, and homogenized in 60 µL of Laemmli SDS-sample buffer in separate Eppendorf tubes using plastic pestles. The samples were centrifuged to remove insoluble debris and then sonicated to sheer nucleic acids. Ten µL from each sample was loaded onto 8% polyacrylamide gels that were then used for Western blotting. The blots were probed with rabbit anti-GFP (cat. TP401 from Torrey Pines Biolabs, Carlsbad, CA, USA) diluted 1/2000, washed, and re-probed with a Vectastain anti-rabbit biotin/avidin-peroxidase kit (Vector Laboratories, Newark, CA, USA). A companion gel with identical loads was stained with Coomassie Blue.

## Figures and Tables

**Table 1:** X chromosome pairs in females used to measure *R2* expression levels in ovaries by RT-qPCR assays presented in Figures 2F and 6).

X Chromosome Pairs	a	b	c	d	e	f	g	h	i	j	k
**a**	***w^1118^***										
**b**	*RpS3/+*		*RpS3/TM2*								
**c**	*TM2/+*										
**d**	*RpL38/+*				*RpL38/CyO*						
**e**											
**f**	*udd^0683-G4^/+*					*udd^0683-G4^/udd^0683-G4^*	*udd^0683-G4^/CyO*	*udd^0683-G4^/udd^Null^*	*udd^0683-G4^/CyO-GFP*		
**g**	*CyO/+*										
**h**	*udd^Null^/+*						*udd^Null^/CyO*		*udd^Null^/CyO-GFP*		
**i**	*CyO-GFP/+*		*RpL14/TM2*								
**j**	*KO121/+*										*KO121/TM3-GFP*

Color key for relative quantifications of *R2* expression normalized to GAPDH: light blue: 0–10, yellow: 10–20, pink: 20–40, and orange: above 40.

**Table 2:** Primer sequences used for RT-qPCR.

Gene	Primer Sequences (5′-3′)	Product Length (bps)
*GAPDH*-Forward	AATTCCGATCTTCGACATGG	91
*GAPDH*-Reverse	GAAAAAGCGGCAGTCGTAAT	
*Tubulin*-Forward	TGTCGCGTGTGAAACACTTC	96
*Tubulin*-Reverse	AGCAGGCGTTTCCAATCTG	
*Nopp140*-Exon 1-Forward	TTTTGTGGGACAAGCGGCTA	154
*Nopp140*-Exon 1-Reverse	TCTGCTGGAAAACCTTGGCT	
*R2*-Forward	AATAGATACGACAGACCGGACATAC	186
*R2*-Reverse	CATAACCTGGACGCTAATGATATGAC	

*R2* RNA probe: LOT PRR247 is proprietary at *Molecular Instruments*.

## Data Availability

The raw data supporting the conclusions of this article will be made available by the authors on request.

## References

[1] Dawid I.B., Rebbert M.L. (1981). Nucleotide sequences at the boundaries between gene and insertion regions in the rDNA of *Drosophila melanogaster*. Nucl. Acids Res..

[2] Eickbush T.H., Eickbush D.G. (2014). Integration, regulation, and long-term stability of *R2* retrotransposons. Microbiol. Spectr..

[3] Long E.O., Dawid I.B. (1979). Expression of ribosomal DNA insertions in *Drosophila melanogaster*. Cell.

[4] Roiha H., Miller J.R., Woods L.C., Glover D.M. (1981). Arrangements and rearrangements of sequences flanking the two types of rDNA insertion in *D. melanogaster*. Nature.

[5] Ye J., Eickbush T.H. (2006). Chromatin structure and transcription of the R1- and R2-inserted rRNA genes of *Drosophila melanogaster*. Mol. Cell Biol..

[6] Fefelova E.A., Pleshakova I.M., Mikhaleva E.A., Pirogov S.A., Poltorachenko V.A., Abramov Y.A., Romashin D.D., Shatskikh A.S., Blokh R.S., Gvozdev V.A. (2022). Impaired function of rDNA transcription initiation machinery leads to derepression of ribosomal genes with insertions of *R2* retrotransposon. Nucl. Acids Res..

[7] Eickbush D.G., Eickbush T.H. (2010). *R2* retrotransposons encode a self-cleaving ribozyme for processing from the rDNA gene locus of *Drosophila melanogaster*. Mol. Cell Biol..

[8] Zhang Q., Shalaby N.A., Buszczak M. (2014). Changes in rDNA transcription influence proliferation and cell fate within a stem cell lineage. Science.

[9] Daiβ J.L., Griesenbeck J., Tschochner H., Engel C. (2023). Synthesis of the ribosomal RNA precursor in human cells: Mechanisms, factors and regulation. Biol. Chem..

[10] He F., James A., Raje H., Ghaffari H., DiMario P. (2015). Deletion of *Drosophila Nopp140* induces subcellular ribosomopathies. Chromosoma.

[11] Baral S.S., DiMario P.J. (2019). The *Nopp140* gene in *Drosophila melanogaster* displays length polymorphisms in its large repetitive second exon. Mol. Genet. Genom..

[12] Waggener J.M., DiMario P.J. (2002). Two splice variants of Nopp140 in *Drosophila melanogaster*. Mol. Biol. Cell.

[13] Meier U.T. (1996). Comparison of the rat nucleolar protein Nopp140 with its yeast homolog SRP40. J. Biol. Chem..

[14] Ginisty H., Amalric F., Bouvet P. (1998). Nucleolin functions in the first step of ribosomal RNA processing. EMBO J..

[15] Chowdhury M.N., Jin H. (2023). The RGG motif proteins: Interactions, functions, and regulations. WIREs RNA.

[16] Ozdilek B.A., Thompson V.F., Ahmed N.S., White C.I., Batey R.T., Schwartz J.C. (2017). Intrinsically disordered RGG/RG domains mediate degenerate specificity in RNA binding. Nucl. Acids Res..

[17] He F., DiMario P.J., Olson M. (2011). Chapter 11: Structure and Function of Nopp140 and Treacle. The Nucleolus. Protein Reviews.

[18] James A., Wang Y., Raje H., Rosby R., DiMario P.J. (2014). Nucleolar stress with and without p53. Nucleus.

[19] Lafita-Navarro M.C., Conacci-Sorrell M. (2023). Nucleolar stress: From development to cancer. Semin. Cell Dev. Biol..

[20] Raje H.S., Lieux M.E., DiMario P.J. (2018). R1 retrotransposons in the nucleolar organizers of *Drosophila melanogaster* are transcribed by RNA polymerase I upon heat shock. Transcription.

[21] King R.C. (1970). Ovarian Development in Drosophila melanogaster.

[22] Iwasaki Y.W., Murano K., Ishizu H., Shibuya A., Iyoda Y., Siomi M., Siomi H., Saito K. (2016). Piwi modulates chromatin accessibility by regulating multiple factors including histone H1 to repress transposons. Mol. Cell.

[23] Khurana J.S., Theurkauf W. (2010). piRNAs, transposon silencing, and *Drosophila* germline development. J. Cell Biol..

[24] Dej K.J., Spradling A.C. (1999). The endocycle controls nurse cell polytene chromosome structure during *Drosophila oogenesis*. Development.

[25] Hammond M.P., Laird C.D. (1985). Chromosome structure and DNA replication in nurse and follicle cells of Drosophila melanogaster. Chromosoma.

[26] Berg C., Sieber M., Sun J. (2024). Finishing the egg. Genetics.

[27] Mahowald A.P., Kambysellis M.P., Ashburner M., Wright T.R.F. (1980). Oogenesis. The Genetics and Biology of Drosophila.

[28] Dapples C.C., King R.C. (1970). The development of the nucleolus of the ovarian nurse cell of *Drosophila melanogaster*. Z. Zellforsch..

[29] Chen H.-K., Pai C.-Y., Huang J.-Y., Yeh N.-H. (1999). Human Nopp140, which interacts with RNA Polymerase I: Implications for rRNA gene transcription and nucleolar structural organization. Mol. Cell Biol..

[30] Miau L.-H., Chang C.-J., Tsai W.-H., Lee S.-C. (1997). Identification and characterization of a nucleolar phosphoprotein, Nopp140, as a transcription factor. Mol. Cell Biol..

[31] Tsai Y.-T., Lin C.-I., Chen H.-K., Lee K.-M., Hsu C.-Y., Yang S.-J., Yeh N.-H. (2008). Chromatin tethering effects of hNopp140 are involved in the spatial organization of nucleolus and the rRNA gene transcription. J. Biomed. Sci..

[32] Marygold S.J., Roote J., Reuter G., Lambertsson A., Ashburner M., Millburn G.H., Harrison P.M., Yu Z., Kenmochi N., Kaufman T.C. (2007). The ribosomal protein genes and *minute* loci of *Drosophila melanogaster*. Genome Biol..

[33] Nelson J.O., Slicko A., Yamashita Y.M. (2023). The retrotransposon *R2* maintains *Drosophila* ribosomal DNA repeats. Proc. Natl. Acad. Sci. USA.

[34] Khipple P., King R.C. (1976). Oogenesis in the female sterile(1)1304 mutant of *Drosophila melanogaster* Meigen (Diptera: Drosophilidae). Int. J. Insect Morphol. Embryol..

[35] Jankovics F., Bence M., Sinka R., Faragó A., Bodai L., Pettkó-Szandtner A., Ibrahim K., Takács Z., Szarka-Kovács A.B., Erdélyi M. (2018). *Drosophila small ovary* is required for transposon silencing and heterochromatin organization, and ensures germline stem cell maintenance and differentiation. Development.

[36] Chong P.A., Vernon R.M., Forman-Kay J.D. (2018). RGG/RG motif regions in RNA binding and phase separation. J. Mol. Biol..

[37] Prasanth K.V., Rajendra T.K., Lal A.K., Lakhotia S.C. (2000). Omega speckles—A novel class of nuclear speckles containing hnRNPs associated with noncoding hsr-omega RNA in *Drosophila*. J. Cell Sci..

[38] Cui Z., DiMario P.J. (2007). RNAi knockdown of Nopp140 induces *Minute*-like phenotypes in *Drosophila*. Mol. Biol. Cell.

[39] Jenkins V.K., Larkin A., Thurmond J., Dahmann C. (2022). The FlyBase Consortium Using FlyBase: A Database of Drosophila Genes Genetics. Drosophila: Methods in Molecular Biology.

[40] Choi H.M.T., Schwarzkopf M., Fornace M.E., Acharya A., Artavanis G., Stegmaier J., Cunha A., Pierce N.A. (2018). Third-generation in situ hybridization chain reaction: Multiplexed, quantitative, sensitive, versatile, robust. Development.

[41] de Cuevas M., Spradling A.C. (1988). Morphogenesis of the *Drosophila* fusome and its implications for oocyte specification. Development.

[42] DiMario P.J., Mahowald A.P. (1987). Female sterile (1) yolkless: A recessive female sterile mutation in *Drosophila melanogaster* with depressed numbers of coated pits and coated vesicles within the developing oocytes. J. Cell Biol..

